# Deciphering mechanisms of brain metastasis in melanoma - the gist of the matter

**DOI:** 10.1186/s12943-018-0854-5

**Published:** 2018-07-27

**Authors:** Torben Redmer

**Affiliations:** 1 Department of Dermatology, Venerology and Allergology, Charité – Universitätsmedizin Berlin, Charitéplatz 1, D-10117 Berlin, Germany; 2 Department of Medical Biochemistry, University of Veterinary Medicine Vienna, Veterinärplatz 1, 1210 Vienna, Austria

**Keywords:** Melanoma, Brain metastasis, Microenvironment, Chemokines, CD271, PI3K/AKT signaling, Checkpoint inhibitors

## Abstract

Metastasis to distant organs and particularly the brain still represents the most serious obstacle in melanoma therapies. Melanoma cells acquire a phenotype to metastasize to the brain and successfully grow there through complex mechanisms determined by microenvironmental than rather genetic cues. There do appear to be some prerequisites, including the presence of oncogenic BRAF or NRAS mutations and a loss of PTEN. Further mediators of the brain metastatic phenotype appear to be the high activation of the PI3K/AKT or STAT3 pathway or high levels of PLEKHA5 and MMP2 in metastatic cells. A yet undefined subset of brain metastases exhibit a high level of expression of CD271 that is associated with stemness, migration and survival. Hence, CD271 expression may determine specific properties of brain metastatic melanoma cells. Environmental cues – in particular those provided by brain parenchymal cells such as astrocytes - seem to help specifically guide melanoma cells that express CCR4 or CD271, potential “homing receptors”. Upon entering the brain, these cells interact with brain parenchyma cells and are thereby reprogrammed to adopt a neurological phenotype. Several lines of evidence suggest that current therapies may have a negative effect by activating a program that drives tumor cells toward stemness and metastasis. Yet significant improvements have expanded the therapeutic options for treating brain metastases from melanoma, by combining potent BRAF inhibitors such as dabrafenib with checkpoint inhibitors or stereotactic surgery. Further progress toward developing new therapeutic strategies will require a more profound understanding of the mechanisms that underlie brain metastasis in melanoma.

**Electronic supplementary material:**

The online version of this article (10.1186/s12943-018-0854-5) contains supplementary material, which is available to authorized users.

## Background

Metastases to the brain are observed in 10–40% of melanoma patients, although the number of metastatic lesions observed in brains post mortem is higher (~ 73–90%), suggesting that most patients develop brain metastases during the course of the disease [1–3]. In 15–20% of melanoma patients, the central nervous system (CNS) is the first site of relapse and is often accompanied by metastases in a second (41%) and third organ (20%) [4]. Currently the cumulative risk at 5 years for patients with melanoma to develop metastases in the CNS is about 7% [5, 6]. In addition, the time to development or detection of melanoma brain metastases ranges from < 1 year to > 5 years [6] with a median time of 2.5 years (30.5 months) [7]. Several risk factors have been identified, including the thickness (Breslow depth > 3 mm [8]), ulceration [7] and the location of the primary melanoma [9].

**Fig. 1:**
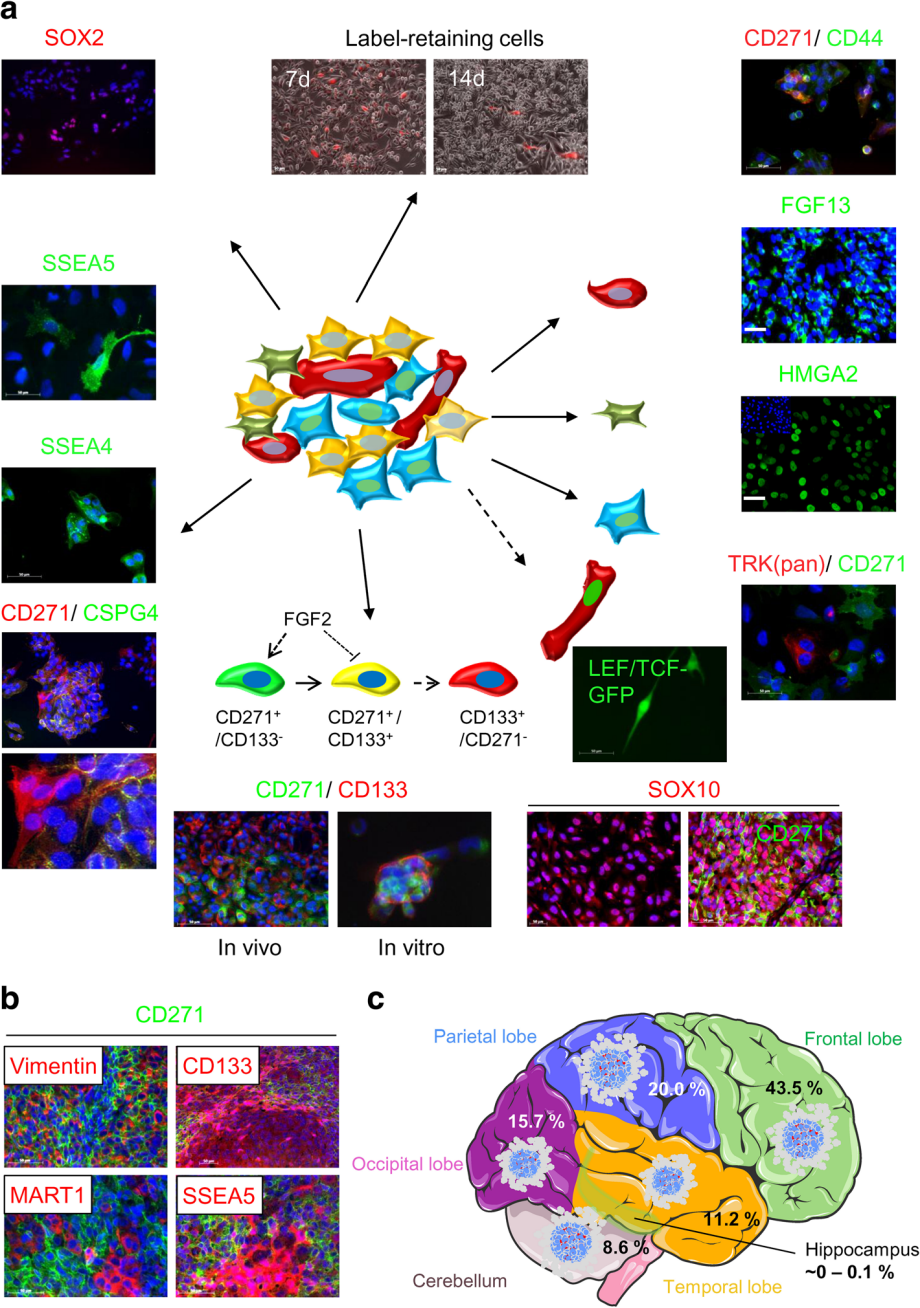
Phenotypical heterogeneity of melanoma cells in vitro. **a** Immunofluorescence microscopy (IF) of melanoma cells revealed only a small number of cells positive for the stage-specific embryonic antigens (SSEA) 4 and 5; as well as slow-cycling cells which retain the lipophilic dye PKH26 (red). Cells with active WNT-signaling, driving expression of a LEF/TCF1-controlled GFP, or cells positive for either CD44 or tropomyosin-related kinases (TRKs) were infrequently observed as well. More frequently observed was the expression of CD271, HMGA2, FGF13, SOX10 (ubiquitous), SOX2, or CSPG4. Cultures of melanoma cells exhibiting co-existing, interconnected phenotypes as shown for CD271 and CD133 are rare. **b** Intratumor heterogeneity, shown by IF of tumors for CD271 (green) and either vimentin, CD133, MART1 or SSEA5 (all red). In (**a**) and (**b**), DAPI served as a nuclear dye. Scale bars indicate 50 μm. **c** Schematic representation of the regional distribution of melanoma brain metastases (BM), numbers represent the frequency of BM observed in 115 patients

Alongside clinical parameters, attempts to identify molecular markers that can predict the dissemination of melanoma cells to the brain have led to the identification of some promising candidates that might permit earlier diagnoses of the disease and generally better prognoses for patient outcomes. But the roles and functions of candidate markers such as cell surface proteins are not clearly understood: do they enhance the capacity of melanoma cells to metastasize to the brain, or are they induced by brain microenvironments and mediate cell survival and proliferation? Generally, a program that initially drives the initial spread of melanoma cells will not necessarily ensure the successful formation of brain macrometastases, as suggested by Fidler et al. [10, 11]. The high plasticity of melanoma cells, demonstrated by an unstable and fluctuating expression of cell surface markers [1–3], may enable cells to respond and adapt to prevailing environmental cues and be a prerequisite for the changes in their fundamental programming (Fig. 1a-b). This suggests that diverse melanoma cells may co-exist in stages with regard to the microenvironment that permit them to interconvert in response to stimuli such as growth factors, chemokines or cytokines [12] and epigenetic cues [13], reviewed in [14] (Fig. 1a). In the light of the diversity normally observed among melanoma cells, metastatic lesions in the brain might be the result of seeding by either a primary tumor or extracranial metastases. Cerebrotropic tumors, particularly desmoplastic neurotropic melanoma, have been reported to exhibit a high brain metastatic capacity [15]. Both scenarios, however, seem to be responsible for intra-tumor heterogeneity and the formation of metastases in the brain from melanoma [16]. A development of extracranial metastases may prime or select for melanoma cells with a higher brain metastatic propensity. This process is probably enhanced by therapeutic interventions and may explain the fact that patients who develop brain metastases during the course of the disease generally experience worse outcomes [17]. Another issue is the spatial and temporal distribution of brain metastases that might follow specific patterns of expression of growth factors. An investigative study of 115 brain metastases [18] revealed that the majority was located within the frontal lobe (43.5%). Brain metastases were less frequently observed in the cerebellum (8.6%) and rarely found in the hippocampus (~ 0–0.1%) (Fig. 1c) [18, 19]. Whether the metastasis to a certain region within the brain is more likely than to other regions remains elusive.

In addition to the timing of the formation of brain metastases (BM), their number and location are of prognostic relevance. Patients with 1 to 3 BM had a median OS of 5.92 months [17]. The outcome of patients with > 3 BM located in the parenchyma was a median of 3.52 months, while metastases located in leptomeninges lead to a median of 1.22 months, respectively [17]. However, this data precede the currently available systemic therapies e.g. the combination of the BRAF inhibitor dabrafenib with trametinib, a very potent inhibitor of MEK1/2. The combination of both inhibitors in the COMBI-MB trial showed an intracranial response and improved OS (10.8 months) [20]. Multiple BM obtained from patients reveal strong inter-patient heterogeneity but a clear intratumor homogeneity [21] regarding the pattern of marker expression within tumors. This suggests a clonal evolution of multiple BM following the establishment of a founder clone or micrometastasis. Recently, Brastianos et al. demonstrated that primary tumors and brain metastases undergo divergent evolutionary paths [22]. Contrary, spatially and temporally separated metastases in single patients’ brains corresponded to each other genetically, again suggesting that multiple BM arise from a single clonal source [22].

**Fig. 2:**
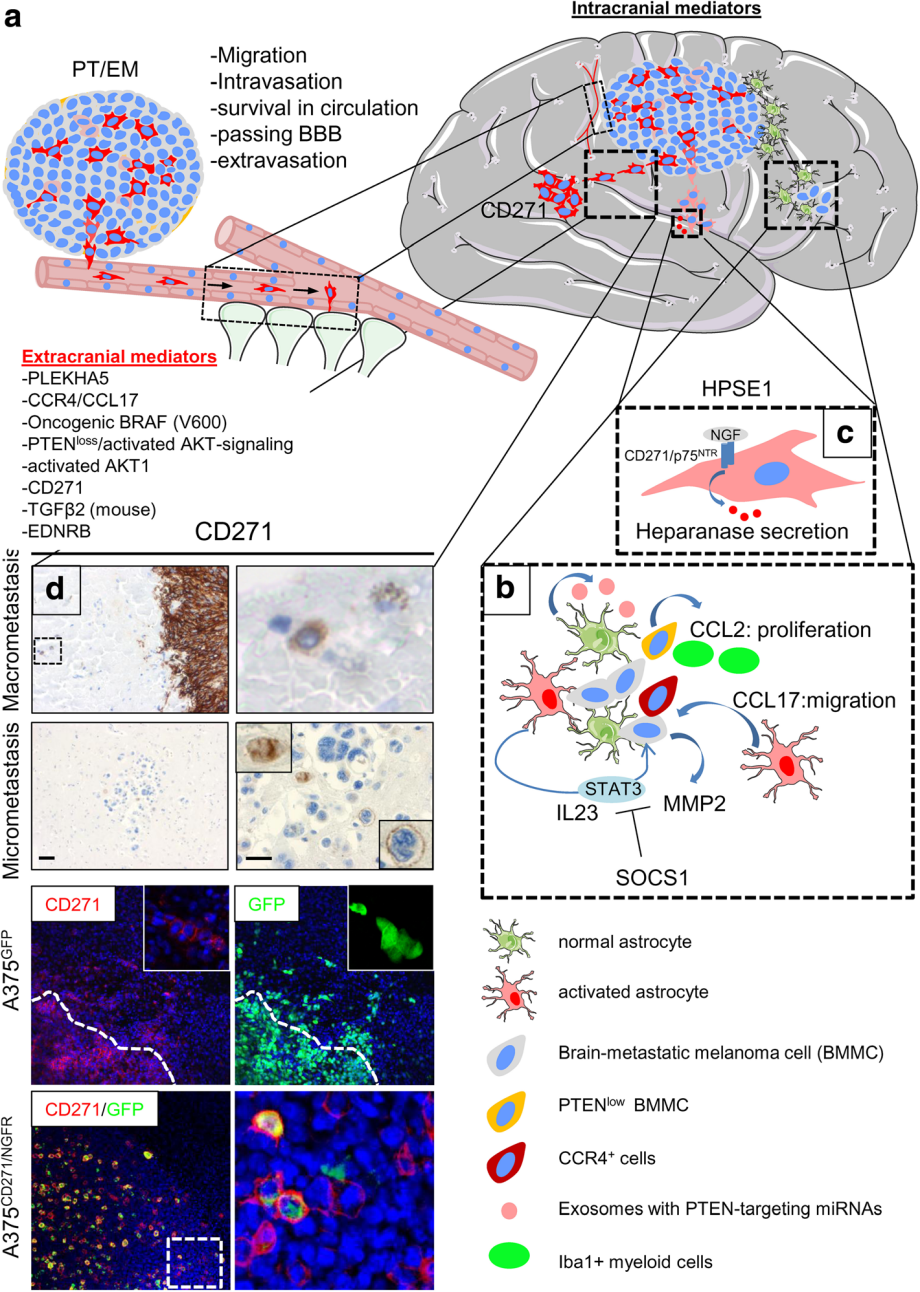
The many routes to brain metastasis for melanoma. **a** Schematic representation of the dissemination of melanoma cells to the brain. Important factors are the Pleckstrin Homology Domain Containing, Family A Member 5 (PLEKHA5), which mediates cells’ transmigration through the BBB and the chemokine receptor CCR4 and its ligand CCL17. CCR4^+^ melanoma cells are probably “homed” to the brain by astrocyte-secreted CCL17; loss of the Phosphatase and Tensin Homolog (PTEN) is frequently observed and leads to hyperactivation of the PI3K/AKT pathway, which can be mimicked by the forced expression of activated AKT1. CD271 may serve as both a “homing” receptor that guides melanoma cells to the brain and a survival factor. Following migration to and intravasation into blood vessels, melanoma cells disseminate either hematogeneously or via lymphatic vessels (not shown). Cells which survive passage through the circulation need to transmigrate through the BBB through a process such as disrupting endothelial tight junctions or secreting of proteolytic enzymes. The BBB is composed of tightly connected endothelial cells which are in close proximity to perivascular endfeet of astrocytes. **b** In the brain, activated astrocytes secrete IL23 or CCL17 or exosome-loaded PTEN targeting miRNAs. The binding of IL23 to the receptor heterodimer IL23R/IL12Rβ1 induces STAT3 phosphorylation and expression of MMP2. Secreted CCL17 attracts CCR4^+^ melanoma cells and CCL17 binding to CCR4 induces the expression of MMP13 through NFkB. The loss of PTEN in brain metastatic melanoma cells induces the secretion of CCL2 which in turn leads to the recruitment of Iba1^+^ myeloid cells. The latter promote the proliferation of brain metastatic melanoma cells. Expression of the negative regulator of STAT-signaling SOCS1 (suppressor of cytokine signaling 1) was down-regulated in melanoma BM. **c** Cerebral migration of melanoma cells is facilitated through the binding of pro-NGF to the NGF-receptor (CD271, NGFR, p75^NTR^) and the secretion of HPSE1. **d** Upper panels: CD271^+^ cells were found in brain micro- and macro-metastases. Lower panels: Brain slice cultures of A375^GFP^ or A375^CD271/NGFR^ cells revealed an extensive migration of melanoma cells with endogenous or stable forced expression of CD271

In principle, tumor cells frequently fail to complete all steps of the metastatic cascade, which leads to a low number of secondary lesions even in the presence of a high proportion of circulating tumor cells [11, 12]. Melanoma cells capable of brain colonization must develop mechanisms that mediate their survival as they circulate and actively transmigrate through the blood-brain-barrier (BBB). The BBB is formed by tightly connected endothelial cells that line cerebral microvessels. The barrier function relies on the presence of tight junction proteins including occludins, claudins and junctional adhesion molecules (JAM), which restrict the passive diffusion of solutes such as therapeutic drugs and small ions from the blood into the extracellular space of the CNS [23], reviewed in [24, 25]. Early studies in quails and chicks revealed that brain endothelial cells physically associate and interact with cells of the parenchyma, most significantly astrocytes, to induce the formation of the blood-brain barrier. This implies that the barrier is not intrinsically regulated by endothelial cells but depends on specific aspects of the brain microenvironment [26]. The perivascular endfeet of astrocytes particularly lie in close proximity to the walls of microvessels in the brain (Fig. 2a), facilitating astrocyte-endothelial signaling and inducing the formation of tighter tight junctions as well as other features of the BBB [24]. Several lines of evidence indicate that melanoma cells adhere to and disturb the interaction of brain endothelial cells through a disruption of tight and adherence junction proteins such as claudin 5 and ZO-1 in a way that facilitates transmigration [27]. In addition, proteolytic enzymes such as heparanase (HPSE1) and seprase (FAP; Fibroblast Activation Protein Alpha) are important for the capacity of metastatic cells to traverse the BBB and occupy the brain [27–29]. Once they have done so, micrometastases give rise to macrometastases through their proliferation along brain microvessels, as demonstrated by Kienast et al. [30], reviewed in [31]. The injection of cancer cells through a chronic cranial window permitted their real-time tracking in vivo using multiphoton laser-scanning microscopy [30]. This revealed that intracerebral seeding of brain macrometastases occurs either passively via the flow of cerebrospinal fluid through the meninges and ventricles or via active migration. The tracking of intracerebrally inoculated melanoma cells also revealed that melanoma cells actively migrate along leptomeningeal and brain parenchymal blood vessels [32].

Here I present recent findings on the mechanisms that drive brain metastasis in melanoma and discuss them in a broader context.

## Current concepts of melanoma brain metastasis

### Oncogene-driven signaling pathways

Oncogenic mutations of the serine-threonine kinase BRAF are found in ~ 40–60% of melanoma and 80% of melanocytic nevi (comprehensively reviewed in [33]), [34]. BRAF mutations are accompanied by amino acid (AA) exchanges of V (valine) to E (glutamic acid), K (lysine), D (aspartic acid) or R (arginine) at position 600 with V600E and V600K as the most prevalent forms (75.4 and 17.2%) [35]. In addition, the frequency of BRAF or NRAS mutations shows a melanoma subtype and metastatic site dependency [36]. Hence, BRAF/NRAS mutations were more frequently observed in lymph nodes or brain sites (62 and 70%) or non-acral cutaneous (51.4%) than acral melanomas (16.2%) [35].

In 2009, Dankort et al. established a mouse model system to decipher mechanisms of melanoma initiation and progression. They found that the expression of constitutively active BRAF^V600E^ under the control of the tyrosinase (TYR)-promoter, which occurs specifically in melanocytes [37], led to melanoma formation only in conjunction with the deletion of the inhibitor of PI3K-activation, PTEN (Phosphatase and Tensin Homolog) [38]. This suggests that neither oncogenic BRAF nor the hyperactivation of PI3K/AKT signaling by PTEN loss [39] is sufficient to initiate melanoma formation alone. The overactivation of PI3K/AKT signaling triggers the activation of the mTOR (mammalian target of rapamycin)-pathway. Given this scenario, the administration of rapamycin ought to reduce melanoma formation, as demonstrated by Stambolic et al. The concerted action of activated AKT1 and BRAF^V600E^ in CDKN2A^loss^ melanoma also promotes lung and brain metastasis [40]. The further loss of PTEN protein expression is significantly correlated with a decrease in overall survival (OS) and a reduction in the time it takes for brain metastases to arise in stage IIIB/C melanoma patients with BRAF (V600) mutations [41]. These data suggest that oncogenic BRAF and activation of PI3K/AKT signaling are probably prerequisites for brain metastasis formation and may occur together. This is supported by the findings of Davies et al., who demonstrated that the activation of PI3K/AKT signaling occurs in distinct ways depending on whether the tumor exhibits an oncogenic mutation in BRAF vs. NRAS. BRAF-mutated tumors expressed low levels of PTEN accompanied by a high activation of AKT through phosphorylation. NRAS-mutated tumors, on the other hand, displayed a normal expression of PTEN but a low level of activated AKT (p-AKT) [42]. However, Davies et al., did not detect a significant difference in CNS involvement among Stage III patients with high levels of p-AKT/low PTEN [42]. Furthermore, a study by Jakob et al. demonstrated that patients with BRAF or NRAS mutated stage IV melanoma show a higher incidence for brain metastases at initial diagnosis as compared to patients carrying none of the mutations [43]. In contrast, the retrospective correlation of clinical parameters with the mutational status of BRAF, NRAS and KIT of 823 patients with melanoma and brain metastases diagnosed between 2006 and 2015 revealed that the time from primary diagnosis to brain metastasis did not vary by mutation and was not associated with survival after the diagnosis of brain metastases [44].

Hence, the information about the presence or absence of a certain mutation in BRAF or NRAS or KIT is not sufficient to predict whether or not a melanoma cell will enter the brain. The consequence of the presence of a specific mutation e.g. in BRAF is very likely determined by other, additional factors e.g. the activation of the PI3K/AKT pathway. The comparison of concordant brain and extracranial metastases revealed that brain metastases have a higher level of activated AKT and a lower expression of PTEN than metastases in the lung or liver [42, 45]. Thus active PI3K/AKT signaling appears to play a pivotal role in brain metastases that arise from melanoma (Fig. 2a-b) and distinguishes them from extracranial metastases while the status of 154 previously reported hotspot mutations was comparable in both matched tumor pairs [46].

This makes it likely that different mechanisms are involved, predetermined by the oncogenic mutation that is present, albeit mutations in passanger genes might also play a role in that process [47, 48]. In addition a comparative analysis of matched pairs of brain and extracranial metastases, using Sequenom mass array-based genotyping, suggested that additional factors determine the fates of systemically disseminated melanoma cells, upon an examination for 154 known hotspot mutations of tumor specimens [46]. Furthermore, Chen et al. observed a high concordance between brain and extracranial metastases and suggested that brain metastasis is encoded in mechanisms that are independent of the mutation or variations in the copy number.

## Signaling processes that mediate brain metastasis - the role of the brain microenvironment

### PI3K/AKT, STAT3 and TGFβ signaling in brain metastasis

Besides mutation-driven, intrinsically activated signaling pathways, a number of studies have demonstrated that the mutations found in extracranial and BM typically match [45, 46, 49]. While the mutational load of the two types of metastases is usually comparable, different signaling pathways are activated, suggesting that soluble factors provided by brain parenchyma cells such as astrocytes are crucial in the establishment of the new tumors. Such cells arise from a glial-restricted progenitor cell, and normally they play roles in the repair of brain tissue and scarring following injuries [50]. Astrocyte-secreted factors include neurotrophins (NGF, BDNF), [51], interleukins (IL-6, IL-8) and G-CSF [52]. These and other factors may be responsible for the hyperactivation of PI3K/AKT signaling in brain tumor cells [45], which would mean that brain parenchymal cells play a crucial role in the establishment and maintenance of BM. Maintenance is partially achieved through the emergence of stem-like cells [21, 53].

Zhang et al. made the intriguing finding that when PTEN is expressed at normal levels in primary tumor cells, its expression is lost after dissemination to the brain, but not to other organs [54]. This mechanism is reversible and relies on interactions between brain metastatic cells and astrocytes, which release exosomes containing PTEN-targeting miRNAs in addition to cytokines and growth factors (Fig. 2b). The transfer of such exosomes to brain metastatic cells lowers PTEN expression while increasing the secretion of CCL2, which in turn leads to the recruitment of Iba1/AIF1^+^ myeloid cells. These reciprocally enhance the survival and proliferation of metastatic tumor cells in the brain [54]. Further evidence for this mechanism was gathered by intracranial injection of an astrocyte-specific Cre-adenovirus into Mirc1tm1.1Tyj/J mice. The adenovirus mediated a knock out of the miR-17-92 allele which is responsible for PTEN repression [55]. The astrocyte-specific ablation of PTEN-targeting miRNAs significantly suppressed brain metastasis of intracarotidly injected B16BL6 cells alongside with a restoration of PTEN and a strong reduction in CCL2 secretion.

To globally dissect stromal-cell driven changes of gene expression in tumors, Park et al. performed microarray-based profiling of highly metastatic and invasive A375SM cells that had been engrafted into the brains of recipient mice [56]. Microarrays with high species-specificity permitted to distinguish changes in gene expression induced by reciprocal interactions of mouse brain parenchyma from those induced by the human tumor cells. Profiling the gene expression in A375SM cells following intracranial transplantation revealed that mouse brain parenchyma cells induced neurological signaling processes including axonal guidance and glutamate receptor signaling. This revealed that in the brain microenvironment, cancer cells acquire the characteristics of the neuronal lineage. This stroma-induced reprogramming of brain tumor cells was also observed in co-cultures with astrocytes. Park et al. suggested that the acquisition of neuronal transcriptional patterns might play a role in the acquisition of chemoresistance among the brain metastases [56]. This is in agreement with a study by Lin et al., which suggested that astrocytes and melanoma cells physically interact via a gap junction-based communication, which in turn mediates chemoresistance among the melanoma cells [57].

### Extracranial signaling processes that mediate brain metastasis

Extracranial metastases from melanomas can be distinguished into two groups: the cerebrotropic tumors, which tend to metastasize early (≤ 6 months) and the non-cerebrotropic tumors which do not form brain metastases (> 18 months) [58]. Jilaveanu et al. observed a high expression of the guanine nucleotide exchange factor PLEKHA5 in cerebrotropic patients and an A375 derivative with a high brain colonizing tendency (A375Br) than in non-cerebrotropic tumors or cells (A375P). In all specimens of tumors from patients, however, PLEKHA5 was high – regardless of the anatomical location [58], albeit the knock-down of PLEKHA5 diminished the proliferation and transmigration of cerebrotropic cells across the BBB in an in vitro model [58]. This suggests that the expression of PLEKHA5 may permit melanoma cells to pass the blood-brain barrier efficiently [59]. PLEKHA5 specifically interacts with phosphoinositides and thus might modulate PI3K/AKT signaling by functionally interfering with mechanisms responsible for their activation [60]. Using the same cell lines, Xie et al. found that the expression of activated (phosphorylated) STAT3 was higher in BM than in primary tumors [61]. Using promoter assays with either a constitutively activated or a dominant negative form of STAT3, they also demonstrated that this transcription factor was responsible for the expression of MMP2, FGF2 and VEGF [61]. This indicated that STAT3-dependent signaling played a role in regulating processes that mediate the invasion and angiogenesis of metastatic melanoma cells to the brain. They also found an association between STAT3 activation and an increase in the number of lung metastases. This suggests that STAT3 activation may be responsible for co-occurrence of lung and brain metastases that has been observed in a study of 216 autopsied tumor specimens [62]. Mechanistically, an elevation in STAT3 signaling was linked to the loss of expression of the suppressor of cytokine signaling-1 (SOCS-1) or IL-23 induced signaling [63, 64]. However, a recent tissue microarray study revealed that the level of activated STAT3 was not associated with an increased risk of developing CNS metastasis or time to CNS metastasis. The study included extracranial metastases of patients who did not have CNS metastasis at the time of the last follow-up. In contrast, STAT3 phosphorylation turned out to be a negative prognostic factor for overall survival (OS) in patients that did not develop CNS metastasis [65].

In addition, the expression of the TGFβ-receptor ligand TGFβ2 seems to play a critical role in site-specific brain metastasis, as demonstrated by Zhang et al. in a mouse model. Patterns of TGFβ2 expression were sufficient to spatially distinguish brain metastases arising from the B16 and K-1735 murine melanoma cell lines. Whereas B16 cells with a low expression of TGFβ2 formed leptomeningeal metastases, overexpressing TGFβ2 in these cells led to the formation of metastases in the brain parenchyma, as was the case for K-1734 cells with high levels of endogenous TGFβ2 [66, 67]. Knocking down TGFβ2 reduced the number of K-1734 metastases to the brain parenchyma. This supports the idea that levels of TGFβ2 play a pivotal role in the spatial distribution of BM, but the precise role of TGFβ signaling in this process has yet to be deciphered for human melanoma. In addition, expression of the G protein-coupled receptor EDNRB (endothelin receptor B) was associated with spontaneous melanoma cell brain metastasis in a murine model system. In addition, the overexpression of EDNRB increased the metastatic aggressiveness and decreased median survival as a result of advanced metastatic disease to lungs [68].

### Chemokines and chemokine receptors in brain metastasis

**Fig. 3:**
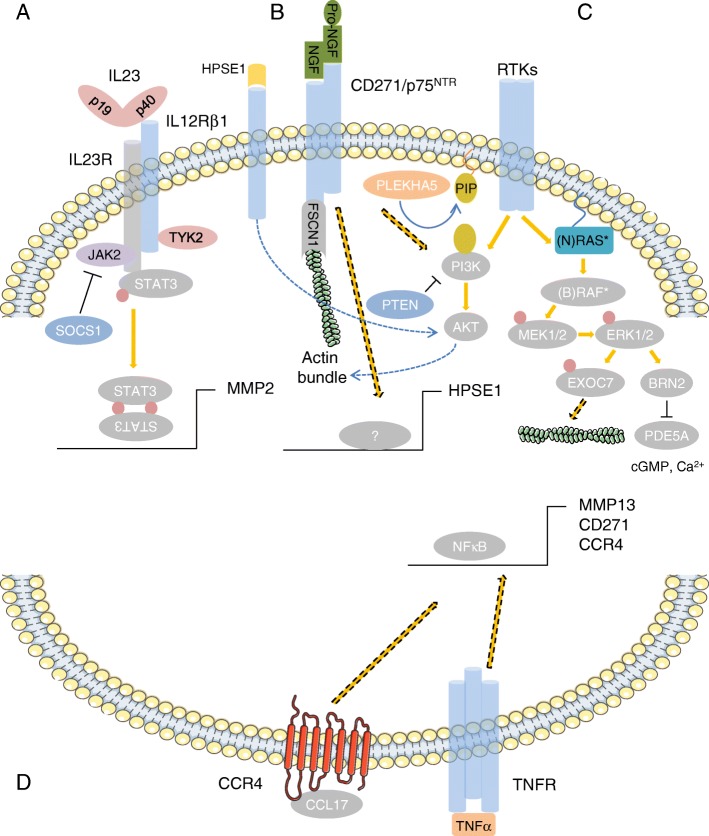
Simplified scheme of signaling pathways and molecules mediating the formation of brain metastases from melanoma. **a** Activation of STAT3 in BM can occur via astrocyte-secreted heterodimeric IL23 and leads to the expression of MMP2. The suppressor of cytokine signaling 1 (SOCS1) binds to and inhibits the kinase activity of Janus-kinase 2 (JAK2). The loss or downregulation of SOCS2 in BM may be responsible for increased stat3 activation. **b** Binding of NGF or pro-NGF induces CD271/p75^NTR^ -mediated FSCN1 binding or HPSE1 secretion or PI3K/AKT signaling via unknown effectors. **c** RTKs induce PI3K/AKT signaling or the RAS/RAF cascade. Mutually exclusive mutant (*) RAS (~ 15–20% NRAS mutations) or RAF (~ 40–50% BRAF mutations) activate the extracellular-regulated kinases ERK1/2, which in turn phosphorylate exo70/EXOC7 (filled red circle), the catalytic subunit of the exocyst complex, or blocks expression of PDE5A via brain-2 (BRN2/POU3F2) to regulate actin dynamics. HPSE1 activity induces AKT-signaling, blocked by PTEN or modified by PLEKHA5. **d** The secretion of CCL17 by astrocytes attracts CCR4^+^ melanoma cells, possibly inducing NFκB-mediated signaling and the regulation of MMP13, CD271 or CCR4 which is in turn regulated via TNFα

Müller et al. identified chemokine receptors and their ligands as mediators of the organ-specific guidance of metastatic cells in breast cancer [69]. Very recently, Klein et al. showed that CCR4 and its ligand CCL17 play a pivotal role in brain metastatic cells derived from melanoma. Forcing the expression of CCR4 in melanoma cells increased their tumorigenicity and the number of brain metastases [70]. This was confirmed in an antibody-based neutralization of CCL17 in vitro, which attenuated melanoma cell migration and transendothelial migration. CCL17 and CCL22, another CCR4 ligand, are secreted by human endothelial cells, astrocytes and microglia. They may serve as chemo-attractants to guide CCR4^+^ melanoma cells to the brain (Fig. 2b) [70], potentially facilitating the formation of multiple BM. This seems to be supported by the fact that the matrix-metalloproteinase 13 (MMP13) is induced by CCR4 in an ERK/NFκB-dependent manner, facilitating invasiveness [71] or migration of colorectal cancer cells through CCR4/CCL17-mediated Rho-kinase signaling [72]. Klein et al. further demonstrated that brain-metastasizing melanoma cells can reprogram astrocytes to express the pro-inflammatory cytokine IL-23. This leads to an up-regulation of MMP2 (Figs. 2b, 3a) and enhanced melanoma cell migration and invasion [73]. Moreover, melanoma micrometastases are also capable of instigating astrogliosis and neuroinflammation, which increases the recruitment of activated astrocytes and further promotes the growth of micrometastatic cells. This seems to be a prerequisite for the establishment of macrometastases [74].

In summary, these data strongly suggest that the brain microenvironment not only attracts a specific subset of melanoma cells but also powerfully determines their fates and subsequent growth, migration and intracranial dissemination. The enhancement of PI3K/AKT, STAT3, TGFβ/SMAD, chemokines and other signaling processes appears to cause a shift in the phenotype of melanoma cells that permits their metastasis to the brain.

## CD271 in melanoma cell migration and brain metastasis

Melanocytes and similar melanoma cells arise from a population of multipotent cells in the neural crest (NC) which normally give rise to healthy neurons, glial cells and astrocytes [75]. Melanoma cells retain the expression of neural crest stem cell (NCSC) genes such as SOX10 [76] and other cellular properties of SC, including plasticity and the capacity to migrate [77, 78]. This tends to support the claim that melanoma cells might be guided (“homed”) by growth factors and cytokines supplied by the brain parenchyma. This was established over a decade ago through studies which demonstrated that the nerve growth factor receptor CD271 (NGFR, p75^NTR^) and receptor ligands such as brain-derived neurotrophic factor (BDNF), nerve growth factor (NGF) and neurotrophin 3 or 4/5 (NTR3, NTR4/5) are involved in the mediation of brain metastases and enhanced survival [79, 80].

Shonukan et al. provided the first evidence that CD271 expression played a role in the migration of melanoma cells, demonstrating that the actin-bundling protein fascin (FSCN1) specifically interacts with CD271 and the actin cytoskeleton in an NGF-dependent manner [81]. This followed work that had been carried out almost a decade earlier by Menter et al., who observed that the expression of the low-affinity p75 neurotrophin receptor (CD271) in human melanoma cells was correlated to their potential to metastasize to the brain [82]. In 1996 Marchetti et al. further demonstrated that treatment with NGF increased the invasive properties of early-passage human brain-metastatic 70 W melanoma cells in vitro but had no effect on the metastasis of melanoma cells to other sites or the behavior of non-metastatic melanoma cells. They traced the heightened invasiveness to increases in the secretion of the proteolytic enzyme heparanase (HPSE1), triggered by pro-NGF and NT-3 (Fig. 2c) [83]. HPSE1 in turn was reported to induce AKT-signaling and probably promote tumor cell migration [84]. Furthermore, an exploration of human melanoma extracranial and BM revealed a predominant expression of CD271 in BM [85].

Most recent explorations of the functional role of CD271 in melanoma have clearly demonstrated that CD271^+^ cells comprise a special subtype of melanoma cells with stem-like properties and a lower expression of melanocyte-markers such as MART1, MITF and tyrosinase [86, 87]. Further support for a crucial role of this receptor in melanoma cell migration came from studies in which CD271 was knocked down, compared to the effects when levels of the endogenous form were high or the molecule was overexpressed [21, 88]. Further studies of BM revealed the presence of migrating CD271^+^ cells in macro- and micrometastases (Fig. 2d, upper panels). Additional evidence came from serial confocal imaging of brain slice cultures of A375^GFP^ cells, which had a high propensity to migrate, and the CD271^+^ cells localized to the migration front (Fig. 2d, center panels). A study tracing A375^CD271/NGFR^ cells clearly demonstrated that cells which stably expressed CD271, hence the level of CD271 was not affected by cellular plasticity, were capable of migration (Fig. 2d, lower panels). These pieces of evidence suggest that melanoma cells very likely express CD271 before colonizing the brain and that this may even be required for their invasive behavior. The stable expression of CD271 enhanced the migratory phenotype even of A375 cells [88], which is intrinsically mediated by oncogenic BRAF via the phosphorylation of cortactin and the exocyst subunit Exo70 [89] and through the ERK1/2-mediated downregulation of the cGMP-specific phosphodiesterase PDE5A [90]. Down regulation of PDE5A in turn increases levels of cGMP and Ca^2+^, regulates actin dynamics and contractility and MMP secretion in melanoma cells. In A375 cells, the migration is probably enhanced by the CD271-dependent expression of genes associated with migration, such as the hyaluronan-mediated motility receptor (HMMR) or fibroblast growth factor 13 (FGF13), which is not secreted but stabilizes microtubules [21, 88, 91].

**Fig. 4:**
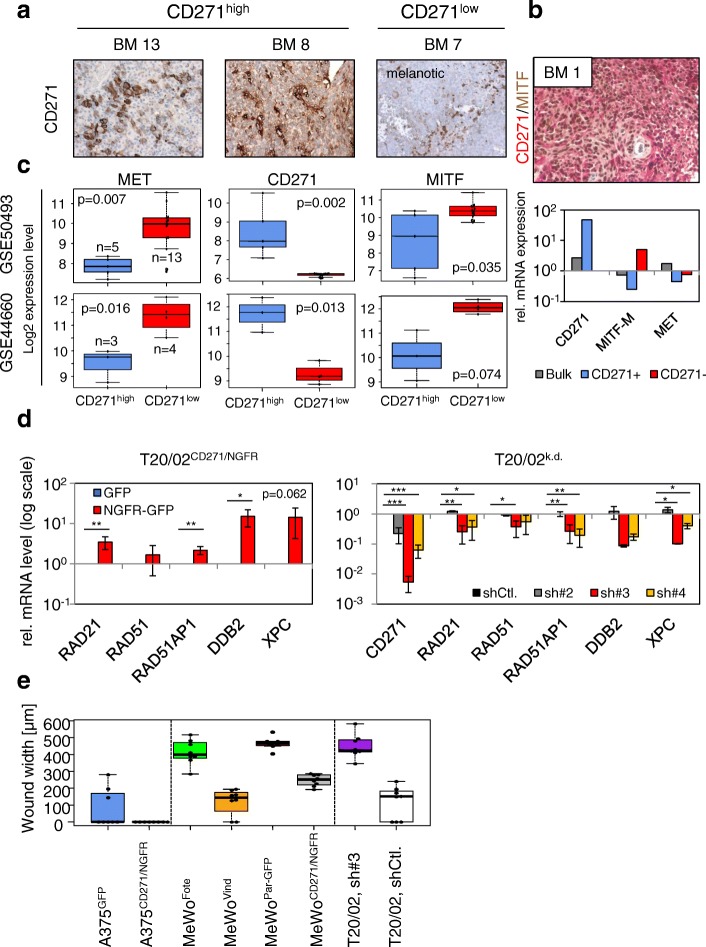
The expression of CD271 determines melanoma cell properties. **a** Expression of CD271 in two representative CD271^high^ brain metastases (BM; 13, 8) as well of a melanotic CD271^low^ BM (7), as determined by immunohistochemistry. **b** Upper panel: Co-staining of CD271 (red) and MITF (brown) of a CD271^high^ BM (1). Lower panel: relative expression of CD271, MITF-M or MET in melanoma cells either positively (CD271^+^) or negatively (CD271^−^) sorted for CD271 or unsorted (Bulk). **c** Levels of expression of MET, CD271 and MITF-M in CD271^high^ and CD271^low^ BM from two independent studies (GSE50493 and GSE44660). *P*-values were determined using the Wilcoxon rank sum test. **d** CD271-dependent regulation of DNA-repair genes in T20/02^CD271/NGFR^ (left panel) or cells stably transfected with CD271-specific shRNAs (#2, #3, #4) by qPCR (right panel). Log2 relative expression levels ± SD of independent triplicates, compared to cells either expressing GFP or knock-down controls (shCtl.) are shown. *p ≤ 0.05; **p ≤ 0.01; ***p ≤ 0.001. **e** Melanoma cells (A375, MeWo) engineered to stably express CD271 and drug-resistant (Fote, Vind) cells show a higher migratory than control cells (GFP). Contrary, the CD271 knock-down (sh#3) strongly decreased cell migration as determined by Live-cell imaging-based scratch-wound assays. Wound closure is indicated by the wound width in μm

Additional work on the BM revealed that a subset of the tumors had high levels of CD271 that clearly distinguished them from a CD271^low^ subset. A comparative analysis of publicly available expression data from BM (GSE50493, GSE44660) revealed that CD271 expression was indeed sufficient to characterize BM. CD271^high^ BM showed a high expression of genes associated with a neural-crest stem-like phenotype and higher levels of DNA-repair genes but a low expression of MITF and its targets among them MET, MLANA or TYR compared to CD271^low^ BM [21] (Fig. 4a-c**,** left panel). Recently, the expression of the melanocyte-specific markers MITF, MLANA and TYR has been associated with a highly proliferative melanoma cell phenotype that metastasizes to multiple organs. The expression of the receptor tyrosine kinase (RTK) AXL, on the other hand, was associated with a type of melanoma cell that was highly invasive but much less proliferative, and predominantly metastasized to the brain [92, 93]. AXL expression was significantly increased in CD271^high^ BM [21], in line with previous observations that these metastatic cells have a slow cycle and proliferate at low rates [78, 94]. Data on the two subtypes suggests that CD271 indirectly regulates mediators of the phenotype of brain-metastasizing melanoma cells (Fig. 4b, lower panel).

## Therapeutic approaches to brain metastases from melanoma

Melanoma patients with brain metastases have a median overall survival (OS) of 2.5–6 months [95] which can be attributed to the difficulty of delivering therapeutic drugs to primary and secondary brain tumors, reviewed in [96], and the fact that BM feature a highly invasive and migratory phenotype [16, 32].

### Systemic therapeutics - chemotherapeutic interventions

Prior to the development of targeted and immunomodulatory therapies, the most clinically relevant approaches to treatment involved chemotherapeutics with dacarbazine (DTIC) as the standard of care for most patients, approved by the FDA in 1975. However, DTIC is incapable of penetrating the blood-brain barrier, hence ineffective in brain metastases [97]. The administration of fotemustine vs. DTIC achieved an improved best overall response rate (BORR) of 15.2% vs. 6.8%. However, the median time to the development of brain metastases was 22.7 months with fotemustine v.s. 7.2 months with DTIC [98]. Switching to TMZ led to high CNS penetrance, but the overall effects remained comparable to those of DTIC [99, 100]. In summary, chemotherapeutic drugs were unable to achieve major improvements in the overall survival of patients; as a result, the current trend has been to replace them with immunomodulatory drugs, particularly for tumors that do not exhibit druggable mutations.

### Targeting therapeutics - immune checkpoint and small molecule inhibitors

Following migration to blood vessels and intravasation, metastatic melanoma cells must escape immune surveillance to survive, extravasate and enter the brain [101]. This evasive capacity is generally mediated by the activation of immune suppressive regulatory T-cells (Tregs) by tumor cells through, for example, the secretion of TGFβ, reviewed in [102]. Tregs accumulate in melanoma, which also exhibit an enrichment of activated CD8^+^ T-cells [103] and the ratio of CD8-positive T cells versus Treg has been found to be a predictor for melanoma patient survival [104]. CD8^+^ T-cells secrete pro-inflammatory cytokines to induce yet another means by which tumor cells evade immune surveillance, involving the up-regulation of programmed cell death-ligand 1 (PD-L1), [103]. PD-L1 binds to the receptor PD-1, expressed on T cells, leading to a reduction in target cell activation [105]. Soluble PD-L1 has been identified as a biomarker for melanoma [106], which hints that a low expression of PD-L1 and PD-L2 might be correlated with favorable patient outcomes. But the expression of PD-1 ligands is restricted to certain melanoma cell subtypes [107]. In the clinic, neutralizing antibodies blocking the interaction of PD-L1 (Pembrolizumab [108], Nivolumab [109]) with PD1, albeit their efficacy remains controversial [110]. A recent study by Goldberg et al. (two-cohort, phase II, clinical trial NCT02085070) revealed that BM from melanoma exhibited partial or complete responses (22%) to pembrolizumab [111]. But the high number of patients whose metastases failed to respond suggests that in many cases, melanoma cells take advantage of additional defense mechanisms that have yet to be identified.

The immune suppression of Tregs is further mediated by their expression of the cytotoxic T-lymphocyte–associated antigen 4 (CTLA-4) and its binding to CD80/CD86, reviewed in [112]). The therapeutic application of the neutralizing antibody ipilimumab yet represents another mechanism of activation of the immune system to fight against tumor cells. The overall survival of patients with visceral metastases (M1c stage) who received ipilimumab was improved (10.1 months) compared to a control group administered a gp100 peptide vaccine (6.4 months) (NCT00094653, Additional file 1: Table S1) [113].

Peritumoral edema is frequently observed alongside with brain metastases formation, leading to increased intracranial pressure and neurological disturbances; symptoms are controlled by administration of corticosteroids [114]. Margolin et al. assessed the efficacy of ipilimumab in patients with either asymptomatic or symptomatic brain metastases who had not or had received corticosteroid treatment at study entry. The study revealed that ipilimumab elicited a disease control in 24% of patients with small and asymptomatic brain metastases, whereas 10% of patients with symptomatic brain metastases, received a disease control within 12 months (NCT00623766, Additional file 1: Table S1, Metadata 1 and 2) [115]. Hence, patients with larger and symptomatic brain metastases who require oral steroids to control peritumoral edema have an ongoing poor response to systemic therapy and are mostly excluded from clinical trials. Therefore, other treatment strategies improving the outcome of these patients are needed e.g. the combination of ipilimumab and the VEGF-neutralizing antibody bevacizumab, a combination which was successfully tested in glioblastoma [116].

Overall, 26% of patients exhibited a refractory response to ipilimumab, and hence might benefit from PD1 inhibitor therapies [108, 117]. A case which has been more comprehensively discussed elsewhere [118, 119].

Strikingly, a dual strategy that used a combination of ipilimumab and nivolumab to block PD1 and CTLA4-mediated immune suppression evoked an intracranial objective response rate of 56%, as demonstrated by Tawbi et al. [120]. And a multicenter US trial (CheckMate204 study, NCT02320058) of melanoma patients with one or more brain metastases achieved a complete response (CR) in 19% of patients; in these cases, the intracranial and extracranial responses largely overlapped. In addition, the survival of patients who received ipilimumab either prior to SRS or thereafter was significantly improved over that of patients who received SRS alone (19.9 months vs. 4.0 months; *P* = 0.009), [121]. Recently, a multicentre open-label randomized phase 2 trial (ABC, NCT02374242, Additional file 1: Table S1) by Long et al. [122] performed in three cohorts of immunotherapy-naive patients revealed an intracranial response to the combination of nivolumab+ipilimumab or nivolumab alone in 46% or 20% of patients with asymptomatic brain metastases with no previous local brain therapy, respectively. The intracranial complete response to nivolumab+ipilimumab or nivolumab alone was 17 and 12%, respectively. However, the intracranial response was markedly reduced (6%) in patients brain metastases in whom local therapy had failed, or who had neurological symptoms, or leptomeningeal disease.

These results clearly demonstrate that therapeutic interventions that block immune suppressive mechanisms are effective in brain metastases, however the identification of strategies for further improving the response to and efficacy of checkpoint inhibitors is mandatory and will provide more insight in the interaction of melanoma and immune cells. A very recent study uncovered the role of the oral and gut microbiome, discriminating melanoma patients who respond and those who do not respond to anti–PD-1 immunotherapy [123]. In addition, since the brain is immune privileged, extracranial and brain metastases very likely feature distinct immune evasion mechanisms. At least for brain metastases, microglia might play an important role. In breast cancer, microglia were associated forced brain metastasis [124].

The identification of mutations of BRAF in human cancers [125] is a milestone, opened new avenues in the therapy of melanoma and was a prerequisite for the development of small molecule BRAF inhibitors, most important vemurafenib and dabrafenib. Besides the high response rate of 53% of patients to vemurafenib accompanied by a median OS of 15.9 months [126], vemurafenib also induces clinical responses in melanoma brain metastases. Albeit, the access of vemurafenib to the brain is restricted by an ABCB1-mediated efflux [127]. Dummer et al. demonstrated in an open-label pilot study that 42% of patients showed an overall partial response (PR) to vemurafenib at both intracranial and extracranial sites, 38% achieved a stable disease and 37% of patients showed a remarkable (> 30%) regression of brain metastases [128]. In addition, a open-label, phase 2, multicentre study of 146 patients with or without previous vemurafenib treatment showed a intracranial BORR (best overall response rate) of 18% as assessed by an independent reviewer committe (IRC). However, 32% or 34% of patients progressed in cohorts without or with previous treatments, respectively [129]. Furthermore, the BREAK-MB trial (NCT01266967 and Additional file 1: Table S1) assessed the response of patients with confirmed BRAFV600E/K mutations to dabrafenib. The study revealed that patients who either had or had not received previous local treatment for brain metastases and progressive brain metastases after previous local treatments showed an intracranial response of 39.2% or 30.8%, respectively. Hence, dabrafenib was effective in brain metastases irrespective of whether they were untreated or have been previously treated and progressed [130]. The intracranial response of patients who either had or had not received previous local treatment for brain metastases was further increased by the combination of dabrafenib and trametinib (56% vs. 58%) in the COMBI-MB study (NCT02039947, Additional file 1: Table S1) [20]. However, the median duration of response was relatively short and might reflect the different modes of activation of signaling pathways and molecular profiles of melanoma brain and extracranial metastases [46].

In summary, targeting therapies have significantly improved the outcome of patients both with extracranial and brain metastases. However, their impact and efficacy might depend on the spatial distribution of metastases.

### Radiosurgery

Single metastases that occur in accessible areas of the brain can be resected or successfully treated with stereotactic radiosurgery (SRS) using gamma or cyber knifes, minimal invasive state-of-the-art techniques which have proven more effective than whole brain radiotherapy (WBRT) in extending OS, with the median reaching 13.9 months [131–133]. Both, WBRT and SRS are associated with severe side effects, most important adverse neurocognitive effects for patients who received WBRT as well as radiation necrosis which is also common but more frequently observed in SRS patients (reviewed in [134]). Radiation necrosis is characterized by fibrinoid necrosis of small arteries and arterioles, likely induced by extensive damage to the vascular endothelium [135].

In a comprehensive clinical study of patients diagnosed with brain metastases from several cancer types except leptomeningeal disease, small-cell lung cancer and hematologic cancer surgical resection followed by SRS of the surgical cavity was proven more effective and significantly lowered local recurrence compared with patients with surgical resection alone (NCT00950001 and Additional file 1: Table S1) [136]. Initially, SRS or the combination of SRS + WBRT was associated with a higher local and distant control than observed for SRS alone, hence adopted to patients with a limited number (1–4) of brain metastases. Albeit this strategy did not improve OS (reviewed in [137]). A multicohort prospective study of 1194 patients with brain metastases of mainly breast and lung cancer, revealed that even in patients with multiple brain metastases (5–10) who had not received previous WBRT [132], SRS was as effective as in patients with 2–4 brain metastases.

To further increase the time to recurrence and improve melanoma patients survival, radiotherapy is combined with targeting drugs or immune checkpoint inhibitors, capable of passing the blood brain barrier. Currently, clinical trials combining ipilimumab and nivolumab with SRS (< 5 progressing BM) or WBRT (≥6 progressing BM) in melanoma patients are in progress (NCT03340129, NCT02097732 and Additional file 1: Table S1). In addition, a recent retrospective study revealed a high (70%) complete or partial response (CR/PR) to concurrent pembrolizumab and SRS [138]. However, the efficacy of these combinatorial trials needs to be proven more comprehensively. Since both extracranial metastases and BM also exhibit a broad response to vemurafenib, dabrafenib and other drugs targeting oncogenic BRAF [20, 129, 139, 140]. Dabrafenib has particularly potent effects on melanoma BM irrespective whether patients had received previous local treatment including surgical resection, WBRT, or SRT and progressed (clinical study NCT01266967) [130].

#### The consequences of drug resistance

Following a positive response towards BRAF-targeting drugs, tumor cells begin to exhibit resistance within 6–7 months [141]. This is marked by an up-regulation of receptor tyrosine kinase receptors PDGFRB [142] or EGFR [143], signaling mediators such as CRAF or NRAS, and the acquisition of mutations in MEK1, MEK2 and NRAS that trigger a stimulation of the RAS/RAF/MAPK pathway (reviewed in [144]). This suggests that melanomas resistant to BRAF inhibitors might be treated with the potent MEK inhibitor trametinib [145]. However, the combination of dabrafenib and trametinib was only moderately effective in patients with BRAF inhibitor–resistant melanoma [146]. Nevertheless, the combination provoked a better response of intracranial BRAFV600-mutant brain metastases from melanoma (clinical trial NCT01266967) compared to monotherapies based on the BRAF inhibitor [130]. At this point another hurdle commonly appeared: cells developed resistance to MEK inhibitors, and the MAPK pathway was reactivated through an acquisition of MEK activating mutations [147] or BRAF gene amplification [148]. Furthermore, two recent studies reported increases in BM or a spontaneous formation of new lesions in patients being treated with vemurafenib [149, 150], most likely due to the mechanisms described above.

### Therapy-induced changes in the expression of markers

Several lines of evidence suggest a therapy-induced enrichment for stem-like tumor cells a mechanism potentially responsible for therapeutic failures and tumor relapse. Kim et al. demonstrated that CD133 -a putative marker of melanoma-initiating cells [151, 152]- acts in concert with the chemokine receptor CXCR4 to facilitate a metastatic phenotype [151]. The expression of CD133 (PROM1), was modified by drug-treatment. Furthermore, in glioblastoma, Bao et al. demonstrated a radiation-induced mechanism responsible for the enrichment of radio-resistant CD133^+^ glioblastoma stem-like cells by activation of the DNA-damage response [153]. In addition, breast cancer-related BM [154] have been associated with a high expression of DNA repair genes and the activation of the PI3K/AKT signaling [155] pathway. These factors may also determine whether radiation therapy will be effective against BM arising from melanoma [156]. Alongside an increased capacity to repair DNA damage, metastatic cells may undergo drug-induced changes in gene expression that help account for their low response to chemotherapies. These therapies in particular induce a DNA damage response in the cells.

The expression of CD271, which is induced by DNA-damaging drugs, may serve as a critical factor in the regulation of DNA repair genes (Fig. 4d) and modifies the migratory phenotype of melanoma cells. Cell migration of melanoma cells was indeed modified in response to the levels of CD271. Hence, increased migration was observed for drug-resistant (Fote, Vind) cells or cells with forced expression of CD271 (NGFR/CD271) [88]. In contrast, the CD271 knock-down was accompanied by a marked reduction in migration [21], (Fig. 4e). Moreover, Lehraiki et al. demonstrated that CD271 promotes vemurafenib-resistance of A375 cells through a NFκB-regulated mechanism. Furthermore, the expression of CD271 was strongly increased in relapsed tumors [157].

Very recently Haueis and Imafuku et al. have reported that melanoma patients undergoing vemurafenib therapy develop more brain metastatic lesions/ tumors than those who do not receive the drug [149, 150]. Along the same lines, Klein and Zubrilov et al. identified CCR4, JARID1B and CD271 among the top up-regulated genes in vemurafenib-resistant, brain metastatic melanoma cells [70, 158]. This raises the provocative question of whether vemurafenib treatment enhances metastasis generally, or is an effect specific to BM in melanoma patients, potentially because the treatment induces the expression of metastasis-promoting factors. Seifert et al. investigated how melanoma metastases responded to vemurafenib in previously drug naive patients. They found that extracranial metastases were more prone to respond completely (CR) or partially (PR) than metastases of the brain or bone. Moreover, the brain was the most common site of progression for patients receiving vemurafenib; among patients who had previously developed brain metastases, 79% experienced a recurrence of BM [159]. The mechanisms responsible for the low response have not been fully elucidated. One suggestion from Seifert et al. is that growth factors from the cerebrospinal fluid (CSF) trigger PI3K/AKT signaling pathways, making BM resistant to vemurafenib [159]. This idea could explain the findings made by Haueis and Imafuku et al.

## Conclusion

Diverse mediators and signaling pathways have been associated with the development of brain metastases in melanoma. While some of the mechanisms that help drive this process seem to be coming into focus, important questions remain: At what point during the disease do metastases develop? Do cells shed from extracranial metastases have a higher propensity to migrate to the brain than primary melanoma cells? If so, what factors might be responsible? A number of studies have suggested that therapeutic interventions frequently seem to have the negative consequence of selecting for cells with a stem-like phenotype. Targeting melanoma cells that express CD133, CD271 or ABCB5 has been shown to block processes of migration [160], metastasis [161] and tumor maintenance [53, 162]. Hence, the combination of targeting small molecule inhibitors and/or immune checkpoint inhibitors with therapeutic interventions to eradicate stem-like cells e.g. siRNA-based strategies may suggest a possible way forward in hopes of further improving patient responses and overall survival.

## Additional file

Additional file 1: **Table S1.** Clinical trials. Results of clinical trials as provided by (https://www.clinicaltrials.gov/) of melanoma patients with brain metastases are summarized. OS = overall survival, PFS = progression free survival, BORR = best overall response rate, assessed by IRC (independent reviewer commitee) is defined as percentage of participants who were responders [with best overall response (BOR) documented as confirmed complete response (CR) or partial response (PR)]. OIR = overall intracranial response, defined as the number of participants whose intracranial response was a confirmed complete response (CR) or partial response (PR) assessed by investigators using modified Response Evaluation Criteria in Solid Tumors (RECIST), version 1.1. **Metadata 1**: provides information about the therapeutic interventions performed within the study as well as the study stage. **Metadata 2**: provides detailed study data particularly drug applications as well as data additional data of NCT01378975, the time to the development of new brain metastases in responders. (ZIP 25 kb)

## Abbreviations

CCL: Chemokine (C-C morif) ligand
CCR: C-C chemokine receptor
CNS: Central nervous system
CR: Complete response
DTIC: Dacarbazine
ERK: Extracellular-signal regulated kinase
Fote: Fotemustine
MAPK: Mitogen-activated protein kinase
OS: Overall survival
PR: Partial response
SRS: stereotactic radiosurgery
Vind: Vindesine
WBRT: Whole brain radiotherapy
