# Supplementary material for: Deciphering mechanisms of brain metastasis in melanoma - the gist of the matter
Source: Mol Cancer. 2018 Jul 27;17:106. doi: 10.1186/s12943-018-0854-5 (PMC6064184; doi:10.1186/s12943-018-0854-5)
Supplement: Supplementary file 1 — Table S1. Clinical trials. Results of clinical trials as provided by (https://www.clinicaltrials.gov/) of melanoma patients with brain metastases are summarized. OS = overall survival, PFS = progression free survival, BORR = best overall response rate, assessed by IRC (independent reviewer commitee) is defined as percentage of participants who were responders [with best overall response (BOR) documented as confirmed complete response (CR) or partial response (PR)]. OIR = overall intracranial response, defined as the number of participants whose intracranial response was a confirmed complete response (CR) or partial response (PR) assessed by investigators using modified Response Evaluation Criteria in Solid Tumors (RECIST), version 1.1. Metadata 1: provides information about the therapeutic interventions performed within the study as well as the study stage. Metadata 2: provides detailed study data particularly drug applications as well as data additional data of NCT01378975, the time to the development of new brain metastases in responders. (ZIP 25 kb) [file 12943_2018_854_MOESM1_ESM.zip › Table S1_Metadata 1.pdf]

NCT Number	Conditions	Interventions	Phases
NCT01515189	Melanoma	Biological: Ipilimumab	Phase 4
NCT00324155	Melanoma	Drug: Ipilimumab|Drug: Placebo|Drug: Dacarbazine	Phase 3
NCT00804908	Melanoma|Metastatic Melanoma|Skin Cancer	Drug: Temozolomide|Drug: ABT-888 (Veliparib) |Other: Placebo	Phase 2
NCT01245062	Melanoma	Drug: GSK1120212 (Trametinib) |Drug: Chemotherapy	Phase 3
NCT01266967	Melanoma and Brain Metastases	Drug: GSK2118436 (Dabrafenib)	Phase 2
NCT01378975	Melanoma and Brain Metastases	Drug: Vemurafenib	Phase 2
NCT00623766	Melanoma and Brain Metastases	Drug: Ipilimumab|Drug: Corticosteroids	Phase 2
NCT01253564	Melanoma and Brain Metastases	Drug: RO5185426 (Vemurafenib)	Phase 2
NCT02230306	Melanoma and Brain Metastases	Drug: Cobimetinib|Vemurafenib	Phase 2
NCT02097732	Melanoma and Brain Metastases	Drug: Ipilimumab|Procedure: Stereotactic Radiosurgery	Phase 2
NCT01781026	Melanoma and Brain Metastases	Drug: Vemurafenib	Phase 2
NCT01721603	Melanoma and Brain Metastases	Drug: Dabrafenib|Procedure: Gamma Knife Radiosurgery|Drug: Trametinib	Phase 2
NCT02039947	Melanoma and Brain Metastases	Drug: Dabrafenib|Drug: Trametinib	Phase 2
NCT02374242	Melanoma and Brain Metastases	Drug: Nivolumab|Drug: Ipilimumab	Phase 2
NCT02320058	Melanoma and Brain Metastases	Drug: Ipilimumab|Drug: Nivolumab	Phase 2
NCT00094653	Melanoma|Metastases	Drug: MDX-010 (anti-CTLA4) monoclonal antibody|Biological: MDX-1379 (gp100) Peptide Vaccine	Phase 3
NCT00587964	Brain Metastasis|multiple entities	Radiation: Stereotactic Radiosurgery	Phase 2
NCT00462982	Kidney Cancer|Melanoma (Skin) and Brain Metastases	Drug: Sunitinib malate	Phase 2
NCT00003308	Kidney Cancer|Melanoma (Skin)|Metastatic Cancer	Radiation: Stereotactic radiosurgery	Phase 2
NCT00039572	CNS Tumors|Melanoma (Skin)|Metastatic Cancer	Drug: boronophenylalanine-fructose complex	Phase 1|2
NCT03325257	Melanoma	Other: Routine care	
NCT02308020	Breast Cancer|Non-small Cell Lung Cancer|Melanoma|BM	Drug: Abemaciclib	Phase 2
NCT01503827	Metastatic Melanoma	Radiation: WBRT	Not Applicable
NCT03075072	Brain Metastases	Radiation: Whole brain radiation|Radiation: Stereotactic radiation (SRS)	Phase 3
NCT02902029	Malignant Melanoma	Drug: Vemurafenib|Drug: Cobimetinib|Atezolizumab	Phase 2
NCT01355120	Ocular Melanoma	Drug: Ipilimumab	Phase 2
NCT02460068	Brain Metastases	Drug: Fotemustine|Drug: Fotemustine and Ipilimumab|Drug: Ipilimumab and nivolumab	Phase 3
