# Supplementary material for: Deciphering mechanisms of brain metastasis in melanoma - the gist of the matter
Source: Mol Cancer. 2018 Jul 27;17:106. doi: 10.1186/s12943-018-0854-5 (PMC6064184; doi:10.1186/s12943-018-0854-5)
Supplement: Supplementary file 1 — Table S1. Clinical trials. Results of clinical trials as provided by (https://www.clinicaltrials.gov/) of melanoma patients with brain metastases are summarized. OS = overall survival, PFS = progression free survival, BORR = best overall response rate, assessed by IRC (independent reviewer commitee) is defined as percentage of participants who were responders [with best overall response (BOR) documented as confirmed complete response (CR) or partial response (PR)]. OIR = overall intracranial response, defined as the number of participants whose intracranial response was a confirmed complete response (CR) or partial response (PR) assessed by investigators using modified Response Evaluation Criteria in Solid Tumors (RECIST), version 1.1. Metadata 1: provides information about the therapeutic interventions performed within the study as well as the study stage. Metadata 2: provides detailed study data particularly drug applications as well as data additional data of NCT01378975, the time to the development of new brain metastases in responders. (ZIP 25 kb) [file 12943_2018_854_MOESM1_ESM.zip › Table S1_Metadata 2.pdf]

NCT Number	Comments	Time to New Brain Metastases in Responders [months]
NCT01515189	*10 mg/kg // **3 mg/kg Ipilimumab	
NCT00324155	*Ipilimumab and Dacarbazine //Placebo and Dacarbazine	
NCT00804908		
NCT01245062	*Trametinib // **Chemotherapy (Dacarbazine 1000 mg/m^2 or Paclitaxel 175 mg/m^2)	
NCT01266967	*150 mg: No Prior Local Therapy // **150 mg: Prior Local Therapy // °BRAF V600E positiv	
NCT01378975	*Previously Untreated Participants // **Previously Treated Participants	14,92*/14,52**
NCT00623766	*Ipilimumab, 10 mg/kg;Corticosteroid-free // **Ipilimumab, 10 mg/kg;Corticosteroid-dependent; °intracranial response	
NCT01253564		
NCT02230306	*median of 4 patients; 150 mg Vemurafenib, 60 m g Cobimetinib	
NCT02097732	*SRS followed 2-3 weeks later by Ipilimumab 3 mg/kg // **Ipilimumab prior SRS	
NCT01781026	Subjects withdrew prior primary outcome measurement therefore no data was obtained to report	
NCT01721603	Dabrafenib/ Trametinib, 150/2 mg, orally; one participant showed a intracranial and overall response	
NCT02039947	*BRAFV600E, asymptomatic BM with no previous local brain therapy; **BRAFV600E, asymptomatic BM with previous local brain therapy ***BRAFV600D/K/R, asymptomatic BM with/without previous local brain therapy; ^BRAFV600D/E/K/R, symptomatic BM, with/without previous local brain therapy	
NCT02374242	*nivolumab+ipilimumab, 1 mg/kg+3 mg/kg; **nivolumab alone; °Patients with asymptomatic brain metastases with no previous local brain therapy ^MEK and BRAF inhibitor therapy-naive patients	
NCT02320058	*Intracranial/**Extracranial	
NCT00094653	*Ipilimumab, 3 mg/kg Plus gp100, 1mg or 2mL // **gp100 1mg or 2mL; °number of participants	
NCT00587964		
NCT00462982	50 mg Sunitinib, °number of participants, ^intracranial response	
NCT00003308		
NCT00039572		
NCT03325257		
NCT02308020		
NCT01503827		
NCT03075072		
NCT02902029		
NCT01355120		
NCT02460068		
